# Continuity and Health Outcomes in Resident Clinics: A Scoping Review of the Literature

**DOI:** 10.7759/cureus.25167

**Published:** 2022-05-20

**Authors:** Margaret J Connolly, William G Weppner, Robert J Fortuna, Erin D Snyder

**Affiliations:** 1 Department of Pulmonary and Critical Care Medicine, University of Rochester Medical Center, Rochester, USA; 2 Department of General Internal Medicine, University of Washington, Seattle, USA; 3 Department of General Internal Medicine, Boise Veterans Affairs Medical Center, Boise, USA; 4 Departments of Internal Medicine and Pediatrics, University of Rochester Medical Center, Rochester, USA; 5 Department of General Internal Medicine, University of Alabama at Birmingham, Birmingham, USA

**Keywords:** internship, residency, resident, continuity of care, continuity

## Abstract

Continuity of care is an essential component of primary care, resulting in improved satisfaction, management of chronic conditions, and adherence to screening recommendations. The impact of continuity of care in teaching practices remains unclear.

We performed a scoping review of the literature to understand the impact of continuity on patients and trainees in teaching practices. A systematic search was performed through PubMed to identify articles published prior to January 2020 addressing continuity of care and health outcomes in resident primary care clinic settings. A total of 543 abstracts were evaluated by paired independent reviewers.

In total, 24 articles met the inclusion criteria and were abstracted by four authors. These articles included a total of 6,973 residents (median = 96, range = 9-5,000) and over 1,000,000 patients (median = 428, range = 70-1,000,000). Most publications demonstrated that higher continuity was associated with better diabetic care (71%, n = five of seven), receipt of preventive care per guidelines (60%, n = three of five), and lower costs or administrative burden of care (100%, n = three of three). A smaller proportion of publications reported a positive association between continuity and hypertension control (28%, n = two of seven). The majority of publications evaluating patient/resident satisfaction demonstrated that better continuity was associated with higher patient (67%, n = four of six) and resident (67%, n = six of nine) satisfaction.

A review of the existing literature revealed that higher continuity of care in resident primary care clinics was associated with better patient health outcomes and patient/resident satisfaction. Interventions to improve continuity in training settings are needed.

## Introduction and background

Continuity of care is a foundational component of primary care that is based on a longitudinal and consistent relationship extending beyond specific episodes of illness or disease [1]. Within non-training settings, better continuity has been associated with improvements in patient and provider satisfaction, management of chronic conditions, adherence to screening recommendations, and decreased emergency department utilization [2-4]. However, less is known about the effects of continuity of care in resident practices where continuity tends to be lower [5].

Improving continuity in resident clinics offers the potential to improve the quality of clinical care and improve resident and patient satisfaction [6]. In addition, improved continuity may enhance the educational experience and lead to better retention in primary care specialties [7].

To more accurately define the effects of improved continuity in resident clinics, we undertook a scoping review to summarize the existing data addressing the influence of continuity in resident primary care clinic settings on measurable patient health outcomes, resident education, patient satisfaction, and cost of care.

## Review

Search process

We conducted a PubMed search using continuity of patient care as a medical subject headings (MeSH) term or any of the following keywords in the title or abstract: COC, continuity, continuity of care, continuity clinic, or continuity clinics. These results were then narrowed to also include internship or residency as a MeSH term or the keywords: resident clinic, residency clinic, or residency clinics. We further limited results to the English language only and excluded case reports, comments, editorials, and letters. We evaluated all studies published prior to January 2020.

All articles identified through the above search process underwent title and abstract review by two independent reviewers to identify all articles that were original research, provided a definition and quantitative value for continuity of care, and focused on continuity in American Council for Graduate Medical Education (ACGME)-recognized residency programs that require a continuity clinic: internal medicine, pediatrics, internal medicine and pediatrics, obstetrics and gynecology, and family medicine. Articles meeting the above criteria were then included in a full-text review by two independent reviewers, searching for a relationship between reported continuity and health outcomes, including quality of care (disease management, receipt of preventive care), costs of care, patient satisfaction, and resident satisfaction. Data abstracted from each article included information on resident, faculty, and patient population; interventions studied when applicable; and continuity measures reported. Any discrepancies between reviewers were discussed with a third reviewer and a consensus was obtained. After abstraction, papers were sorted based on outcomes reported, and descriptive statistics were used to compare results reported.

Definitions

Common definitions used to quantify continuity of care in included studies are as follows:

Usual provider of care (UPC): UPC is the number of visits a patient had with his or her primary care provider (PCP) as a percentage of the total number of the individual patient’s visits. This can range from zero (no primary care visits by the patient with their assigned PCP) to 100% (all visits with their assigned PCP) [8-10].

Continuity for physician (PHY): PHY is the number of appointments a physician had with his or her assigned patients as a percentage of the total number of the individual provider’s scheduled appointments. This can range from zero (no primary care clinic slots of the resident filled with their assigned patient) to 100% (all clinic slots filled with their assigned patients) [11].

Modified modified continuity index (MMCI): MMCI is the total number of physicians (including the PCP) seen by an individual patient divided by the total number of visits for a single patient; it emphasizes dispersion between providers. This can range from zero (each visit with a different provider) to one (all visits with the same provider) [8,12].

Continuity of care index (COC): The COC index factors in the frequency of visits to each provider, as well as the dispersion of visits among them. It can range from zero to one [8-10].

Results

**Figure 1:**
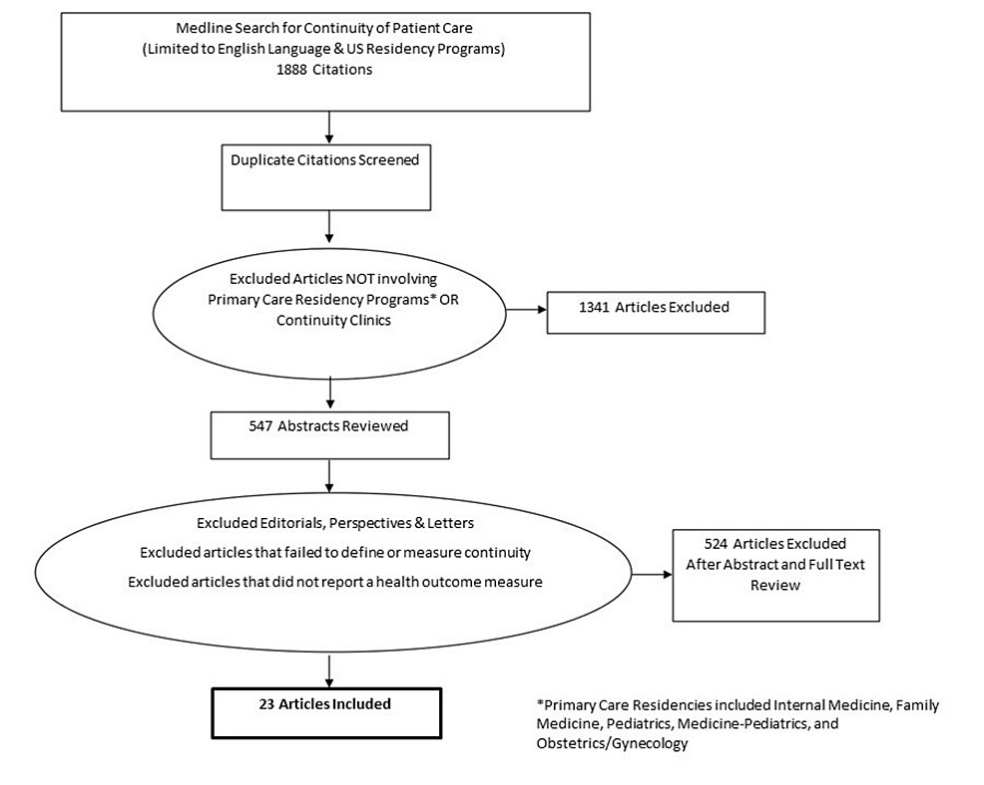
PRISMA diagram for study selection. PRISMA: Preferred Reporting Items for Systematic Reviews and Meta-Analyses

A total of 543 studies were initially reviewed for possible inclusion (Figure 1). In total, 23 articles met all the inclusion criteria and reported a health or satisfaction outcome. These 23 articles included a total of 6,997 residents (median = 96, range = 6-5,000) and over 1,000,000 patients (median = 459, range = 70-1,000,000). The median study duration was 22 months (range = one week-four years). The most commonly reported measure to quantify continuity of care was UPC, which was specifically measured and reported in 52% of publications. The Median measured baseline UPC in resident continuity clinics was 59% (range = 43-75%).

Of the 23 articles included in the full-text review, 10 (43%) reported on health outcomes, 13 (57%) reported on patient and/or resident satisfaction, and three (13%) reported on costs of care.

Health Outcomes

Most publications concluded that higher continuity in residency practices was associated with better health outcomes (Table 1). Commonly evaluated health outcome measures included diabetic care (hemoglobin A1c, regular foot and eye examinations, and nephropathy screening), hypertension control, and compliance with preventive care recommendations, particularly cancer screening.

**Table 1:** Overview of studies: clinical care. UPC: usual provider of care; MMCI: modified modified continuity index; LDL: low-density lipoprotein

Study	Design	Setting	Participants	Duration	Outcome
Diabetic care					
Parchman and Burge [13]	Retrospective, cross-sectional survey	A network of six family medicine residencies	76 family physicians, 397 patients	Six months	Process measures higher for patients who had seen their usual provider once in the last year. Quality of care associated with UPC (r = 0.148, p = 0.03)
Dearinger et al. [14]	Retrospective chart review	Internal medicine residency	83 internal medicine and internal medicine-pediatrics residents, 15 faculty preceptors, 70 patients	Three years	Significant relationship between change in HbA1c and resident UPC (p = 0.02). 94% of patients in the top quartile UPC had improved A1c
Nguyen et al. [15]	Retrospective chart review	Internal medicine residency	38 faculty physicians and 96 internal medicine residents, 650 patients	Two years	Resident patients had more missed appointments. More frequent missed appointments associated with higher HbA1c. Lower continuity contributed to more missed appointments
Younge et al. [16]	Retrospective chart review and diabetes registry	Family medicine residency	484 patients	Two years	No association between MMCI and diabetes quality of care process measures (80% of patients in the sample achieved process measures). There was a relationship between continuity and HbA1c control (low continuity and higher HbA1c)
Wieland et al. [17]	Retrospective pre-post scheduling intervention	Internal medicine residency	96 residents	Two years	Continuity decreased with intervention. No change in quality metrics (HbA1c, LDL, blood pressure, microalbumin)
Fortuna et al. [18]	Retrospective, cross-sectional survey	Four training programs (internal medicine, pediatrics, family medicine, internal medicine-pediatrics) and 30 affiliated non-teaching practices	140 residents, 66 faculty, 134 community physicians, 117,235 visits	One year	Higher continuity was associated with achieving HbA1c < 8%
Jantea et al. [19]	Retrospective pre-post scheduling intervention	Internal medicine residency	208 residents, 39 core faculty		Visit continuity decreased after intervention, no change in HbA1c
Hypertension					
Fisher et al. [20]	Retrospective longitudinal cohort	Family medicine residency	459 patients with hypertension	Two years	No association between continuity and systolic or diastolic blood pressure, or controlled vs. uncontrolled. Non-significant trend to improvement with increased continuity when evaluated by tertiles
Dearinger et al. [14]	Retrospective chart review	Internal medicine residency	83 internal medicine and internal medicine-pediatrics residents, 15 faculty preceptors, 70 patients	Three years	No association between continuity and blood pressure
Nguyen et al. [15]	Retrospective chart review	Internal medicine residency	38 faculty physicians and 96 internal medicine residents, 650 patients	Two years	Resident patients had more missed appointments. More frequent missed appointments associated with uncontrolled blood pressure. Lower continuity contributed to more missed appointments
Younge et al. [16]	Retrospective review of chart and diabetes registry	Family medicine residency	484 patients	Two years	No association between continuity and blood pressure control
Wieland et al. [17]	Retrospective pre-post scheduling intervention	Internal medicine residency	96 residents	Two years	Continuity decreased with intervention. No change in quality metrics (HbA1c, LDL, blood pressure, microalbumin)
Fortuna et al. [18]	Retrospective, cross-sectional survey	Four training programs (internal medicine, pediatrics, family medicine, internal medicine-pediatrics) and 30 affiliated non-teaching primary care practices	140 residents, 66 faculty, 134 community physicians, 117,235 visits	One year	Higher continuity associated with achieving blood pressure of <140/80 mmHg
Jantea et al. [19]	Retrospective pre-post scheduling intervention	Internal medicine residency	208 residents, 39 core faculty		Visit continuity decreased after intervention, no change in blood pressure
Preventative care					
Angelotti et al. [21]	Retrospective pre-post intervention to institute patient-centered medical home care	60 teaching hospitals (118 residencies in internal medicine, family medicine, pediatrics, internal medicine-pediatrics)	5,000 residents, >1,000,000 Medicaid beneficiaries	Four years	Improvements over baseline seen for breast cancer screening, colon cancer screening, tobacco use screening and cessation counseling
Neiderman et al. [22]	Retrospective cohort, pre-post integrated child behavior specialist	Pediatrics residency	363 patients	Three years	Continuity improved with intervention. No difference in immunization rates
Nguyen et al. [15]	Retrospective chart review	Internal medicine residency	38 faculty physicians and 96 internal medicine residents, 650 patients	Two years	Resident patients had more missed appointments. More frequent missed appointments were associated with less preventative care. Lower continuity contributed to more missed appointments
Wieland et al. [17]	Retrospective pre-post scheduling intervention	Internal medicine residency	96 residents	Two years	Continuity decreased with intervention. No change in preventative care obtained
Fortuna et al. [18]	Retrospective, cross-sectional survey	Four training programs (internal medicine, pediatrics, family medicine, internal medicine-pediatrics) and 30 affiliated non-teaching primary care practices	140 residents, 66 faculty, 134 community physicians, 117,235 visits	One year	Higher continuity patients more likely to have colon and breast cancer screening

Diabetic care: The majority of publications (71%; n = five of seven) demonstrated that better continuity was associated with improved diabetic care. The largest and most direct comparison between diabetes control and continuity examined the relationship between continuity and diabetes control in a single internal medicine residency practice [14]. Within this study, the average UPC was 43%, with a range of 11-100%. The authors found that a UPC of 65% or higher was associated with better diabetic control and a 10% decrease in hemoglobin A1c over the two-year study period. A total of 94% of patients at this continuity level showed some improvement in hemoglobin A1c. A second study evaluating resident primary care practices demonstrated a positive relationship between both PHY and UPC and better diabetes control [18]. A third study did not show a consistent relationship between process measures and continuity, perhaps because over 80% of patients had already received the recommended care [16].

Hypertension: Several studies evaluated the relationship between continuity and hypertension with mixed results. Two of seven (28%) publications assessing hypertension demonstrated that better continuity was associated with improved hypertension control. The largest study examined several internal medicine residency programs with wide variation in continuity and found that those residents who achieved higher continuity measured by PHY achieved better blood pressure control for their patients [18]. Another study showed that patients with a higher proportion of missed visits, used as a surrogate for continuity, were more likely to have at least two blood pressure measurements >160/100 (hazard ratio (HR) = 4.36, confidence interval (CI) = 2.51-7.44) [15]. A study within a university-based family medicine practice showed a trend toward better blood pressure control in the highest continuity tertile compared to the lowest; however, this trend did not reach statistical significance (-0.2 mmHg, p = 0.2) [20]. Four other studies failed to show a statistically significant association between continuity and blood pressure control [14,16,17,19].

Preventive care: Five studies reported on the association between continuity and preventive care service delivery, again with mixed results. One study showed that both PHY and UPC were related to timely receipt of colon and breast cancer screening [18]. Similarly, a study in a family medicine practice showed that patients who missed more appointments were less likely to be up to date in preventive care [15]. In another study, authors evaluated the transition to a new scheduling model [17]. They did not see a change in preventive care services (defined as receipt of cervical cancer screening, bone density testing, and lipid screening in eligible patients) despite a decrease in continuity after the intervention.

Two studies assessing the impact of continuity on preventive care evaluated systematic changes in care delivery. The first described the integration of Healthy Steps into a pediatric residency program [22]. This program integrated an early childhood development specialist within the clinic, including presence during the clinic visit. While patients enrolled in this program did see an improvement in continuity (COC 0.24 vs. 0.11), there was no change in immunization rates for enrolled patients. The second study described the transition of 60 teaching hospitals in New York State to incorporate patient-centered medical home practices [21]. Most resident practices were not able to measure continuity before the transition, as patients and providers were not empaneled. However, others [23,24] showed that empanelment alone can improve continuity in a resident clinic. After the pilot, programs reached reasonable resident continuity (PHY = 55%) and demonstrated improvement in breast cancer screening (47% vs. 64%, p = 0.01) and colon cancer screening (48 vs. 59%, p < 0.001), as well as tobacco screening and cessation counseling (70 vs. 86%, p < 0.001).

Patient Satisfaction

The majority of publications (67%, n = four of six) evaluating patient satisfaction suggested that better continuity was associated with higher patient satisfaction (Table 2) [19,25-28]. Most studies utilized external patient satisfaction surveys [17,23,29,30], although two developed their own surveys [24,25]. One study found that although patients generally felt that urgent issues could be dealt with by a non-assigned provider, those with the lowest continuity were the most dissatisfied. In the same study, continuity was particularly correlated with satisfaction in patients who had high numbers of visits (>10/year) [24]. In another study in which a scheduling change showed a decrease in continuity, patient satisfaction was not impacted; however, the continuity was 64% at baseline and decreased to 51%.

**Table 2:** Overview of studies: cost of care and patient and resident satisfaction.

Study title	Design	Setting	Participants	Duration	Outcome
Costs of care					
Rulin et al. [24]	Retrospective chart review, pre-post scheduling intervention	Obstetrics-gynecology residency	Nine residents, 164 patients	34 months	Residents and staff reported that continuity promotes more efficient use of physician time, fewer tests ordered, and results in better overall patient care
Christakis et al. [2]	Retrospective claim review	Pediatric residency	759 patients	Four years	Unadjusted data showed higher continuity patients were less likely to visit emergency department. When fully adjusted, only faculty continuity was associated with emergency department use
Neher et al. [31]	Retrospective chart review, pre-post scheduling intervention	Family medicine residency	Eight faculty physicians and 24 family medicine residents, 1,709 patients	Two years	Continuity improved with intervention. Staff time required to schedule appointments decreased by 75%
Patient and resident satisfaction					
Blankfield et al. [26]	Prospective chart review	Family medicine residency	19 residents, four faculty	Three months	Continuity correlated with multiple item scores for resident satisfaction with clinic. Continuity explains half of the variance in physician satisfaction with practice
Belardi et al. [28]	Prospective pre-post scheduling intervention	Family medicine residency	Six residents, six faculty	15 months	Intervention improved continuity. Residents reported increased satisfaction with office practice because of increased continuity. No change in patient satisfaction
Morgan et al. [23]	Retrospective chart review	Family medicine residency	36 residents, 276 patients	One week	Not seeing one’s provider over multiple visits was associated with lower satisfaction and less likely to recommend center. Those most dissatisfied had worst continuity (four percent). High-usage patients (>10 visits/year) were most dependent on continuity for satisfaction
Warm et al. [29]	Retrospective chart review, pre-post scheduling intervention	Internal medicine residency	108 residents, 489 patients	Three years	Intervention improved continuity. Residents reported higher satisfaction scores, and value of continuity experience was higher. Patient satisfaction scores improved with intervention
Wieland et al. [20]	Retrospective pre-post scheduling intervention	Internal medicine residency	96 residents	Two years	Continuity decreased with intervention. No change in resident satisfaction, but improvement in the ability to focus on clinic and perceived inpatient/outpatient balance. Patient satisfaction did not change
Tuli et al. [21]	Prospective cohort, scheduling intervention	Pediatric Residency	31 residents, eight faculty	Four months	Continuity improved with intervention. Resident satisfaction did not change. Patient satisfaction improved after intervention
Francis et al. [32]	Cross-sectional review	12 internal medicine residencies	713 residents	Nine months	Continuity was not associated with resident satisfaction or patient satisfaction

Resident Satisfaction

Resident satisfaction was evaluated through a variety of measures including direct surveys of resident satisfaction (Table 2). The majority of studies (63%, n = seven of 11) demonstrated that better continuity was associated with higher resident satisfaction [19,25-30]. One publication with a resident survey demonstrated an association between higher continuity and higher resident-reported personal reward from work, a greater sense of relationship with patients, and increased ownership of patient care [30]. The remaining publications showed no change in resident satisfaction with improved higher continuity of care.

Cost of Care

Only three publications evaluated the impact of higher continuity of care on the cost of care (Table 2). The three endpoints evaluated were the frequency of emergency department (ED) visits, the number of tests ordered, and staff time to schedule appointments.

One retrospective study of 164 patients in an obstetrics and gynecology residency practice found that nurses surveyed after reviewing patients’ charts felt that better continuity resulted in fewer diagnostic tests being ordered and improved resident efficiency [28]. In a second publication, researchers retrospectively reviewed claims data of 785 pediatric Medicaid patients and found that higher continuity was associated with decreased ED utilization when comparing high to low continuity patients, although this association was limited to attending providers, and was not seen with resident providers [2]. The third study described a transition to a scheduling system that allows for more clinic sessions per week. Continuity increased and staff time to schedule appointments decreased by 75% [13].

Discussion

In this review, most publications demonstrating higher continuity in resident clinics were associated with better patient care outcomes, as well as resident and patient satisfaction. This is consistent with other studies focused on non-resident providers, suggesting that increased continuity in resident primary care clinics is similarly associated with improved outcomes [2-4].

Control of diabetes had the most frequent association with continuity. Control of hypertension and delivery of preventive care services was less likely to show a consistent association with continuity. Delivery of preventive care services may be less related to a relationship with a particular primary care physician. A continuous therapeutic relationship between patient and clinician may be more important to care for chronic diseases such as diabetes, which requires significant patient engagement and lifestyle change.

Higher levels of continuity of care were associated with both higher patient and resident satisfaction. One important study demonstrated an association between continuity and higher resident-reported personal reward from work, a greater sense of relationship with patients, and increased ownership of patient care [13]. These findings are particularly relevant to attenuating residency burnout and supporting a meaningful clinical experience for trainees and patients.

Maximizing continuity in resident clinics can be incredibly difficult given the competing demands of training [11]. However, one study found several high-performing practices in which continuity approached or exceeded levels in the community practices [17]. These high-performing practices shared several common factors, including consistent use of scheduling protocols, more faculty time in the clinic, lack of advanced practice providers, rescheduling policies when clinics are missed, and dismissal policies for patients with excessive missed appointments [17]. Most of these are clinic-level decisions and could be undertaken without significant change in resources.

Patient empanelment, the concept that a patient has a clearly defined primary care provider, was often lacking in the studies of resident practices. Empaneling resident patients is critical to accurately measuring continuity. In fact, this one act may significantly improve continuity as it signals to all team members the importance of returning a patient to his or her primary resident [23].

Once empaneled, resident time in the clinic seems to have the largest impact on continuity [31-34]. Given a stable panel size, more appointment availability will increase UPC because the same number of patients will have more possible spaces to schedule with their primary residents. Increasing panel size while holding appointment availability stable will increase PHY as the resident will have more primary patients to fill his or her appointments. Improving UPC is more directly correlated with improved patient outcomes [5]. As a result, emphasizing greater time in the clinic, rather than adjusting patient panel size, may be more effective in improving quality.

The appropriate goal for continuity in resident clinics is not clear. The Veterans Health Administration previously established a national benchmark for continuity that “three out of four times” the patient sees his or her primary care provider [35]; this would be equivalent to a UPC of 75%. Typically, levels of continuity in resident clinics are lower than those in non-trainee practice settings. In a recent review, resident continuity clinics had a median UPC of 56%, while comparable faculty UPC metrics ranged from 63% to 78% [5]. This emphasizes the need for great focus on improving continuity in resident clinics.

Strengths of this study include a systematic approach and a large sample size (n = 24) of articles from varied residency continuity clinic settings. Limitations include heterogeneous study design with some interventions and some cross-sectional analysis, varied evaluation of outcomes, and frequent lack of adequate controls in the experimental studies. As a scoping review, this work is intended to broadly review the literature for an association between continuity in training clinics and health outcomes and to generate hypotheses for future study [36]. We did observe some heterogeneity regarding the impact of continuity on the outcomes studied based on the study design. Longitudinal or retrospective studies were more likely to demonstrate an association [2,13-16,18,20,24,25,27,37,38], whereas evaluation of an outcome in response to an intervention was more likely to show no association [17,19,21-23,26,28-31].

## Conclusions

Similar to findings in non-resident physician clinics, our review of the literature suggests that significant benefits may be gained from efforts aimed at improving continuity of care in resident clinics. Some of the strongest associations are for patient and resident satisfaction, but other clinical and utilization benefits appear to have important associations as well, most notably in diabetes care. More studies with appropriate controls are needed to establish benchmarks for reasonable expectations for continuity among residency clinics and to identify the most effective methods for achieving improved continuity, given the unique challenges in resident continuity clinic scheduling.

## Appendices

**Table 3:** Preferred Reporting Items for Systematic reviews and Meta-Analyses extension for Scoping Reviews (PRISMA-ScR) Checklist. JBI = Joanna Briggs Institute; PRISMA-ScR = Preferred Reporting Items for Systematic reviews and Meta-Analyses extension for Scoping Reviews *Where sources of evidence (see the second footnote) are compiled from bibliographic databases, social media platforms, and websites. † A more inclusive/heterogeneous term used to account for the different types of evidence or data sources (e.g., quantitative and/or qualitative research, expert opinion, and policy documents) that may be eligible in a scoping review as opposed to only studies. This is not to be confused with information sources (see the first footnote). ‡ The frameworks by Arksey and O’Malley (6) and Levac and colleagues (7) and the JBI guidance (4, 5) refer to the process of data extraction in a scoping review as data charting. § The process of systematically examining research evidence to assess its validity, results, and relevance before using it to inform a decision. This term is used for items 12 and 19 instead of “risk of bias” (which is more applicable to systematic reviews of interventions) to include and acknowledge the various sources of evidence that may be used in a scoping review (e.g., quantitative and/or qualitative research, expert opinion, and policy document). From: Tricco AC, Lillie E, Zarin W, O'Brien KK, Colquhoun H, Levac D, et al. PRISMA Extension for Scoping Reviews (PRISMAScR): checklist and explanation. Ann Intern Med. 2018;169:467–473. doi: 10.7326/M18-0850 [36].

Section	Item	PRISMA-ScR Checklist item	Reported on page #
Title			
Title	1	Identify the report as a scoping review	1
Abstract			
Structured summary	2	Provide a structured summary that includes (as applicable): background, objectives, eligibility criteria, sources of evidence, charting methods, results, and conclusions that relate to the review questions and objectives	2
Introduction			
Rationale	3	Describe the rationale for the review in the context of what is already known. Explain why the review questions/objectives lend themselves to a scoping review approach	4
Objectives	4	Provide an explicit statement of the questions and objectives being addressed with reference to their key elements (e.g., population or participants, concepts, and context) or other relevant key elements used to conceptualize the review questions and/or objectives	5
Methods			
Protocol and registration	5	Indicate whether a review protocol exists; state if and where it can be accessed (e.g., a Web address); and if available, provide registration information, including the registration number	n/a
Eligibility criteria	6	Specify characteristics of the sources of evidence used as eligibility criteria (e.g., years considered, language, and publication status), and provide a rationale	5
Information sources*	7	Describe all information sources in the search (e.g., databases with dates of coverage and contact with authors to identify additional sources), as well as the date the most recent search was executed	5
Search	8	Present the full electronic search strategy for at least 1 database, including any limits used, such that it could be repeated	5
Selection of sources of evidence†	9	State the process for selecting sources of evidence (i.e., screening and eligibility) included in the scoping review	5
Data charting process‡	10	Describe the methods of charting data from the included sources of evidence (e.g., calibrated forms or forms that have been tested by the team before their use, and whether data charting was done independently or in duplicate) and any processes for obtaining and confirming data from investigators	5
Data items	11	List and define all variables for which data were sought and any assumptions and simplifications made	5 and table of articles
Critical appraisal of individual sources of evidence§	12	If done, provide a rationale for conducting a critical appraisal of included sources of evidence; describe the methods used and how this information was used in any data synthesis (if appropriate)	n/a
Synthesis of results	13	Describe the methods of handling and summarizing the data that were charted.	5
Results			
Selection of sources of evidence	14	Give numbers of sources of evidence screened, assessed for eligibility, and included in the review, with reasons for exclusions at each stage, ideally using a flow diagram	Figure 1
Characteristics of sources of evidence	15	For each source of evidence, present characteristics for which data were charted and provide the citations	Tables 1, 2
Critical appraisal within sources of evidence	16	If done, present data on critical appraisal of included sources of evidence (see item 12)	n/a
Results of individual sources of evidence	17	For each included source of evidence, present the relevant data that were charted that relate to the review questions and objectives	6-11
Synthesis of results	18	Summarize and/or present the charting results as they relate to the review questions and objectives.	6-11
Discussion			
Summary of evidence	19	Summarize the main results (including an overview of concepts, themes, and types of evidence available), link to the review questions and objectives, and consider the relevance to key groups	11-13
Limitations	20	Discuss the limitations of the scoping review process	13
Conclusions	21	Provide a general interpretation of the results with respect to the review questions and objectives, as well as potential implications and/or next steps	13-14
Funding			
Funding	22	Describe sources of funding for the included sources of evidence, as well as sources of funding for the scoping review. Describe the role of the funders of the scoping review	N/A
